# Quantum Simulation of Fractal Fracture in Amorphous Silica

**DOI:** 10.3390/ma18153517

**Published:** 2025-07-27

**Authors:** Rachel M. Morin, Nicholas A. Mecholsky, John J. Mecholsky

**Affiliations:** 1 Physics Department and Vitreous State Laboratory, The Catholic University of America, Washington, DC 20064, USA; mecholsky@cua.edu; 2 Department of Materials Science and Engineering, University of Florida, Gainesville, FL 32611, USA; jmech@mse.ufl.edu

**Keywords:** brittle fracture, amorphous silica, fractal geometry, computational simulations, AM-1 method, quantum mechanics, COSMO method, reconfiguration fracture energy, multiscale fracture, fractal fracture

## Abstract

In order to design new materials at atomic-length scales, there is a need to connect the fractal nature of fracture surfaces at the atomic scale using quantum mechanics modeling with that of the experimental data of fracture surfaces at macroscopic-length scales. We use a semi-empirical quantum mechanics simulation of fracture in amorphous silica to calculate a parameter identified as a critical characteristic length, $a_{0}$, which has been experimentally derived from the fractal nature of fracture for many materials that fail in a brittle matter. To our knowledge, there are no known simulation models other than our related research that use the fractal parameter $a_{0}$ to describe the fractal fracture of the fracture surface using quantum mechanical simulations. We provide evidence that $a_{0}$ can be calculated at both the atomic and macroscopic scale, making it a fundamental property of the structure and one of the elements of fractal fracture. We use a continuous random network model and reaction coordinate method to simulate fracture. We propose that fracture in amorphous silica occurs due to bond reconfiguration resulting in increased strain volume at the crack tip. We hypothesize two specific configurations leading to fracture from a four-fold ring reconfiguration to three-fold ring or (newly observed) five-fold ring configurations resulting in a change in volume. Finally, we define a reconfiguration fracture energy at the atomic level, which is approximately the value of the experimental fracture surface energy.

## 1. Introduction

Although fracture in materials is a critical problem in many systems, there is still not a fundamental understanding of the fracture process that unites the atomic and macroscopic-length scale behavior [1,2]. One of the fundamental questions in fracture is as follows: What is the atomic-length scale origin of the fracture process? The second question is as follows: Can the characteristic length be scaled to macroscopic-length scales? A bridge across length scales of fracture can improve the way materials are designed to resist failure. To better understand the fracture process, both experimental and computational efforts have been attempted at many length scales [2,3]. To our knowledge, there are no other studies which involve calculations of bond breaking or reconfiguration using quantum fracture as it is related to the fracture surface in macroscopic fracture. The only studies that have fractal fracture modeling in the literature are for crack branching conditions [4,5,6,7,8,9]. We have shown in previous research [10] that fracture surface fractal dimensions are different from the fractal dimensions in the crack branching scenarios. The fractal dimension on fracture surfaces is a material constant related to the energy required for fracture and is independent of strength. The fractal dimensions in crack branching conditions are different for each strength level. To avoid confusion, we have previously termed the latter fractal dimension the “Crack Branching Coefficient” [10]. What is needed is a methodology that unites the fracture process at all length scales. The application of fractal geometry to the fracture process leads to the identification of a common parameter $a_{0}$ that unites these scales. From a materials design perspective, $a_{0}$ can be used to predict the fracture toughness of materials from its atomic structure [10]. Already, $a_{0}$ has been calculated computationally in previous work and has been shown to be of the same order of magnitude as experimental values [1]. It is the purpose of this present work to extend these semi-empirical molecular-orbital computational results from single rings to larger structures in order to verify that $a_{0}$ is a link between length scales of fracture.

### 1.1. Fractal Nature of Fracture

Fractal geometry, common in nature, has been used to describe the shape of clouds, coastlines, dielectric breakdown, and soot aggregates [11]. Tortuous fracture surfaces also exhibit self-similarity, or self-affinity, at multiple length scales, which is evidence of its fractal nature [12,13,14]. Many studies have shown that fracture is a fractal process [1,10,15,16,17,18,19,20,21,22,23,24].

At the micro- and macro-scale, linear elastic fracture mechanics offers a methodology for describing the fracture process related to crack size, *c*, and applied stress at fracture, σ:

(1) $$\sigma =\frac{K_{IC}}{Yc^{\frac{1}{2}}}=\sqrt{\frac{2E\gamma }{Y^{2}c}}$$

where $K_{IC}$ is the critical stress intensity factor, *Y* is a geometric constant of loading and crack shape, *E* is the elastic modulus, and γ is the fracture energy [25,26]. Since the fracture process is fractal in nature, there should be a relationship between the fractal dimension of the fracture surface and the properties shown in Equation (1). Experiments have been performed to measure the $D^{*}$ for many materials and shown to correlate to $K_{IC}$ and γ [27,28,29,30,31,32]. In fact, the relationship has been shown to be

(2) $$K_{IC}=E{\left(a_{0}D^{*}\right)}^{\frac{1}{2}}$$

or

(3) $$\gamma =\frac{1}{2}Ea_{0}D^{*}$$

where $a_{0}$ is a material structural parameter, and $D^{*}$ is the fractal dimensional increment [33]. The measurement of $D^{*}$ is related to the tortuosity of the fracture process and is averaged over the fracture surface [34]. The fracture energy γ is the energy required to propagate a crack in a material. It has SI units of J/m^2^ and can be thought of as the energy needed to open a unit of area in the material. The constant of proportionality between $D^{*}$ and γ must have units of energy per square length, and it has been hypothesized to be related to the elastic modulus of the material, *E*, which has units of energy per length cubed. What remains in the proportionality constant is a parameter with units of length, which has been named $a_{0}$ [1].

The critical relation depends on $a_{0}$, which is a considered to be a structural parameter whose experimental values have been found to depend on the material class [1]. The parameter $a_{0}$ has been related to the stretched bond length during fracture for single crystals [35], as well as the free volume created at the crack tip for glasses [1]. The crack tip is assumed to be a series of broken bonds within a continuous random network of atoms. Experimentally, this has been determined for many materials that fail in a brittle manner [22]. The parameter $a_{0}$ can be determined in quantum mechanics simulations by obtaining the strain at fracture. The details of the calculations are provided in Section 2.5.

### 1.2. Computational Fracture at the Atomic Scale

**Figure 1:**
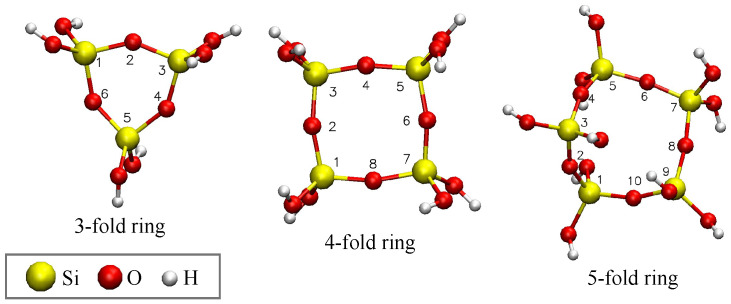
Three-, four-, and five-fold silica rings, with atoms in the ring labeled by indices. The geometry is optimized using AM-1 [37]. Visualization: VMD [42].

A computational method for finding $a_{0}$ at the atomic scale has been hypothesized with results that have the same order of magnitude as experimental data [1,22]. Specifically, for vitreous silica, SiO_4_ ring structures were simulated [36] using the Austin Model-1 (AM-1) [37], a semi-empirical molecular orbital approximation to the solutions of the Schrödinger wave equation. Ring structures in vitreous silica can contain three, four, five, or more SiO_4_ tetrahedra, bonded together in a closed loop [38,39,40,41], as seen in Figure 1.

Starting with a 3-, 4-, or 5-fold ring, the geometry was optimized using the AM-1 method [37]. Geometry optimization in this case consists of finding the arrangement of atoms that minimizes the heat of formation ($H_{f}$) of the molecule. The structure after fracture was found using a reaction path method [43]. The product, or end-state, of the fracture process was unknown and was predicted by performing a coordinate scan. In a coordinate scan, the length of a bond or the degree of an angle between atoms is changed in increments, and the geometry is optimized at each step with a constraint on the scanned coordinate. In [36], a single Si-O bond in the ring was extended by 0.01–0.02 nm at each step to simulate the crack tip during the fracture process. After the bond was extended by 0.1–0.3 nm, a sudden re-configuration of the molecule occurred, and a new 3-, 4- or 5-fold ring was formed, corresponding with a drop in $H_{f}$. This reconfiguration and drop in $H_{f}$ were interpreted to be the point of fracture of the ring. The difference between the initial $H_{f}$ and its value just before it dropped was labeled as the fracture barrier, which is the energy needed to be supplied to the molecule in order to cause the reconfiguration of the geometry. It is assumed that lower fracture barrier (or $H_{f}$ barrier) means that the ring requires less energy to break. It was observed that the most energetically favorable reaction path was a ring-contraction event, where 4-fold or 5-fold rings reconfigured into 3- or 4-fold rings, respectively. The 4-fold ring had the lowest fracture barrier of 77 kcal/mol, whereas the 5-fold and 3-fold rings had slightly higher fracture barriers of 103 and 96 kcal/mol, respectively [36]. The parameter $a_{0}$ was calculated geometrically by measuring the initial diameter of the ring and the strain fraction of the severed Si-O-Si bond before and after ring contraction to get an estimate of the free volume created at the crack tip. The success of these computational $a_{0}$ calculations, which were repeated for four different materials, is that the values have the same order of magnitude as experimental data. The theoretical connection between $a_{0}$ and the free volume created at the tip of the crack is explained in [1,34]. The limitation of these computations was that they used single-ring structures to represent the fracture process on the surface of a bulk material. It remains to answer whether this ring-contraction phenomenon is seen in larger networks of amorphous silica and if calculations of $a_{0}$ produce similar values for these larger structures.

### 1.3. Calculating $a_{0}$ in Larger Structures of Amorphous Silica

In this present study, we extend the work of West et al. [1,36] to determine if ring contraction is observed in the fracture of larger structures and to calculate $a_{0}$ for these structures. We focus on amorphous silica (a-silica) using the continuous random network (CRN) model, which is an idealized theory for the structure of amorphous solids [41,44]. The model should have some short-range order, including ring structures as seen with Raman spectroscopy [39,40].

**Figure 2:**
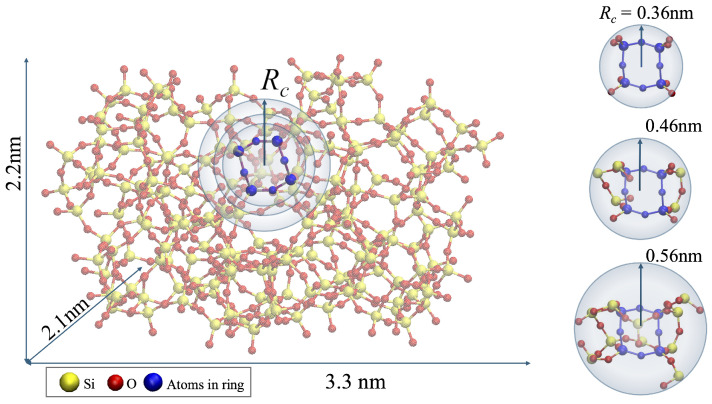
Amorphous silica model with coordinates from [45]. One of the 4-fold rings is highlighted in blue. The various clipping radii $R_{c}$ are shown as blue arrows and a blue circle around the chosen ring. Various structures clipped from the model are shown on the right with their respective $R_{c}$.

An existing CRN model, developed by Bell and Dean [38], with accessible digitized atomic coordinates [45], was used for our molecular simulations, as seen in Figure 2. The model fits the radial distribution function (RDF) of vitreous silica well, and it has 4-, 5-, and 6-fold ring structures. Though a few methods for automatically creating amorphous structures have been developed, such as the melting and quenching of silica in MD simulations [46], or the Monte-Carlo bond-switching method [47], the Bell and Dean model displays a comparable accuracy to the structures produced by these methods. In addition to its accuracy and accessibility, another advantage of Bell and Dean’s model is that it is a well-known and specific structure so that we can concentrate on the local behavior for the quantum calculations of the fracture process.

To perform the fracture simulations, we wanted to keep the accuracy of quantum-based methods while still being able to model larger structures with more than 16 heavy atoms, which is the number of O and Si atoms in a four-fold ring, using fractal analysis as a guide. We considered using density functional theory (DFT) to model the fracture process. However, modeling fracture processes of large systems with DFT is complicated by the high-dimensionality of the potential energy landscape. The more atoms that are included in a structure to be optimized, the more variables that are involved in the geometry relaxation. DFT calculations, which scale as O($N^{3}$) where *N* is the number of atoms, are more computationally expensive than semi-empirical methods, which scale as O($N^{2}$). While DFT promises more robust results, the flip-side is that it cannot simulate fracture for more than a few atoms. Most fracture experiments with DFT have focused on crystalline materials and at most deform the structures in the elastic response regime to determine Young’s Modulus [48,49,50,51,52,53]. One group went beyond this limit by introducing a separation distance between crystalline planes, 1–4 atoms each, relaxing the structure at each distance, and reached non-convergence in the geometry relaxation calculation right at the point of fracture [54]. Fracture has been simulated with DFT via static loading in structures with up to four atoms [51], but gaps in DFT’s capabilities to simulate fracture in larger structures still need to be addressed.

Molecular dynamics is at the opposite end of the spectrum in that it can handle large systems but at the expense of high approximation. It has been used extensively in fracture studies, even to the point of multi-million atom simulations [55]. However, there are still issues with molecular dynamic simulations of atomistic fracture [51], which produce effects that have not been proven experimentally [3].

In order to simulate larger structures, while still maintaining an acceptable accuracy of quantum mechanics, semi-empirical molecular orbital models were utilized in this present study. In order to compare the results with West et al. [1,36], the same parameterization, AM-1, was used. This present work pushes semi-empirical simulations of fracture to their limit. Whereas before these methods were limited by computational resources, now we have reached the limit of the potential energy landscape and the ability for the calculation to converge.

In this present work, we simulate larger structures of amorphous silica by identifying SiO_4_ rings in Bell and Dean’s model and selecting a certain range of atoms in the surrounding network. Next, we calculate $a_{0}$ using the same theory and method as was used by West et al. [1], adapted to larger structures. We then compare the fracture phenomena (ring contraction) and measurements of $a_{0}$, along with the $H_{f}$ barriers, at each structure size. Additionally, we define a reconfiguration fracture energy by dividing the $H_{f}$ released during fracture by the change in surface area of the resulting geometry of the molecule after fracture. We compare the reconfiguration fracture energy to the fracture energy as defined by Griffith [56] and the related strain energy release rate as defined by Irwin [57].

## 2. Methods

### 2.1. Model

Four-fold rings, which have the lowest fracture barrier according to [36], were chosen to be at the crack tip in the simulated system. There are 48 four-fold ring structures in the Bell and Dean model. Increasingly larger structures are simulated by selecting a single four-fold ring and including more and more of the atoms in the model surrounding the ring, using a clipping radius. In this study, three of the four-fold rings were chosen. The clipping radius, $R_{c}$, determines the size of a sphere of atoms around one of the rings in the model. The atoms outside that sphere are “clipped” off and not included in the simulated structure. The clipped structures are so small, at most 1 nm in diameter, that it makes little difference whether a ring is chosen from the surface or the center of the model. An $R_{c}$ of 0.36 nm will give only the atoms in the four-ring plus some singly coordinated O atoms bonded to the Si atoms. The clipping radii ranged from 0.3 to 0.5 nm, and the number of atoms per selection ranged from 24 to 66. Clipping radii are chosen so that subsequently larger structures have one or more additional Si atoms attached in a continuous network to the 4-fold ring. Examples of molecules extracted from the Bell and Dean CRN model using different $R_{c}$ are shown in Figure 2.

After selecting the atoms within the sphere, the molecule is cleaned up and prepared for simulation by completing each of the SiO_4_ tetrahedra and adding H atoms to singly-coordinated O atoms. This is necessary so that the valencies of the atoms are satisfied and that the molecule’s net charge is neutral. Any atoms that are not attached by any sequence of bonds to the original four-ring of interest are removed. Next, the initial molecule’s geometry is optimized using the AM-1 method. This prepares the molecule for a transition state coordinate scan.

### 2.2. Program Details

The focus of this research is fast fracture, where the molecule’s interaction with the environment is minimal, so the molecule was simulated in a vacuum. As in reference [36], AM-1 was chosen as the main theory. Calculations were performed on Molecular Orbital PACkage (MOPAC) software, using openmopac version 22.1.1. Calculations were repeated using both restricted Hartree-Fock (RHF) and unrestricted Hartree–Fock (UHF) orbitals, with no significant differences between the results when the “Precise” command, which tightens the optimization criteria, was used. The program automatically uses an eigen-following (EF) method for molecules with less than 30 atoms and L-BFGS is used for molecules with 30 or more atoms. The molecule’s structure was input into the program using the Z-matrix syntax. The ordering of the atoms in the Z-matrix matters, and a random ordering will produce nonphysical correlations between atoms in the molecule [58]. In this study, the atoms were arranged in the Z-matrix so that the bond distances between atoms in the matrix were minimized. This made it so that the bond lengths between adjacent atoms were optimized during the simulation, rather than between atoms that were not directly bonded.

### 2.3. Coordinate Scans

The fracture process is simulated using a coordinate scan, which increases the bond distance between two atoms in a molecule in steps of a given length and optimizes the geometry of the rest of the molecule at each step. In this case, coordinate scans are performed on each of the Si-O bonds in the four-ring of interest with a step size of 0.01 nm for about 50 steps. During the coordinate scan, the heat of formation of the molecule is calculated at each step. Initially, $H_{f}$ will increase as the two atoms are separated. The geometry is optimized at each step to find the configuration that will give the lowest $H_{f}$ possible, given the constraint of the Si-O bond distance. At a certain point, $H_{f}$ will drop suddenly, and the molecule’s geometry will reconfigure to a local energy minimum. At this point, usually a new ring is formed with the atoms from the original four-ring of interest. For example, a four-fold ring could rearrange into a three-, four-, or five-fold ring. All eight Si-O bonds in each of the three 4-fold rings was scanned individually for each clipping radius.

### 2.4. Identifying the Point of Fracture

During a coordinate scan, the point of fracture is identified by (1) a drop in $H_{f}$, (2) a reconfiguration of the geometry, and (3) a creation of free volume in the model by the formation of new structural units, usually rings.

**Figure 3:**
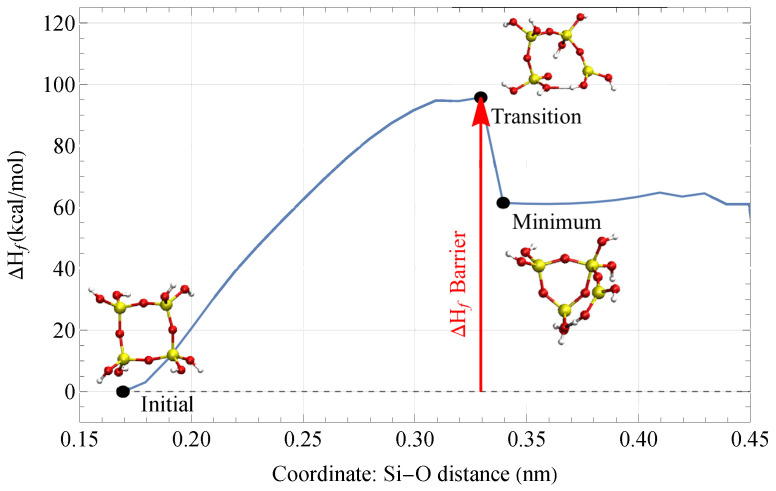
Change in the heat of formation $H_{f}$ of a four-fold ring during a coordinate scan. When $H_{f}$ drops, the four-fold ring contracts into a three-fold ring.

A drop in $H_{f}$ signifies that the molecule has reached a local minimum in its configuration energy landscape. Using the same terminology as transition-state search methods, the geometry at a $H_{f}$ peak just before it drops is the transition structure. The $H_{f}$ barrier is the difference between $H_{f}$ at the transition state, right before the drop, and its initial state, as seen in Figure 3. The $H_{f}$ barrier is interpreted to be the fracture barrier when the conditions for the fracture are met. The fracture barrier is the amount of energy needed to put into the system in order to initiate a fracture.

It is assumed that the fracture occurs when the molecule reaches a local energy minimum in its reaction path, given the constraint of the fixed coordinate, i.e., the distance between the Si and O atoms involved in the crack tip. The drop in $H_{f}$ is accompanied by a significant reconfiguration of the geometry of the molecule, where some bonds are broken and formed. In a-silica, the bond rearrangement leads to the formation and destruction of SiO_4_ tetrahedra-ring structures. The reorganization of the geometry that creates volume will lead to fracture. Thus, a change in volume is required to simulate the fracture event. The quantity $a_{0}$ is an indirect measurement of that free-volume creation.

### 2.5. Calculating the Structure Parameter

**Figure 4:**
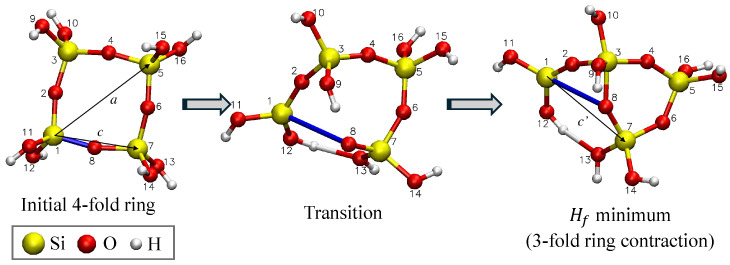
Example of a coordinate scan where a 4-fold ring contracts into a 3-fold ring after fracture. The coordinate bond length between atoms 1 and 8, shown in blue, is incrementally increased by 0.01nm with geometry optimization at each step. The distances *a*, *c*, and $c^{\prime }$ are used to calculate $a_{0}$ using Equations (4) and (5).

After fracture, the structure parameter $a_{0}$ is calculated using the method from [1]. According to this method,

(4) $$a_{0}=\frac{a}{\epsilon }$$

where *a* is the greatest diameter of the initial ring before straining, and ϵ is the strain fraction between the two silicon atoms in the severed Si-O-Si bond before and after fracture. Given that *c* is the initial distance between the Si atoms, and $c^{\prime }$ is the distance between the same Si atoms after the molecule reconfigures (see Figure 4), ϵ is defined as

(5) $$\epsilon =\frac{c^{\prime }-c}{c}$$

The method is an indirect measure of the free volume created during the fracture process, where the free volume is proportional to $\epsilon^{3}$ [59].

### 2.6. Reconfiguration Fracture Energy

In order to compare the change in the heat of formation during fracture with existing experimental measurements, we calculate the reconfiguration fracture energy, $\gamma_{r}$, by the following procedure:

1. After a coordinate scan, outlined in Section 2.3, the energy released $\Delta H_{f}$ is calculated from the difference between the transition $H_{f}$ and the minimum $H_{f}$ after fracture.
2. The COSMO area [60] is then calculated for the initial and post-fracture geometries.
3. $\Delta H_{f}$, with units of kcal/mol, is converted into kJ/molecule.
4. $\gamma_{r}$ is estimated to be the $H_{f}$ per molecule released divided by the change in COSMO area per molecule.

We call the resulting value the reconfiguration fracture energy because it is the energy per unit area released after a reconfiguration of the molecule’s geometry, which leads to fracture.

## 3. Results

### 3.1. Results of Coordinate Scans

We conducted a total of 152 coordinate scans of single-bond lengths in amorphous silica networks, starting from about 0.17 nm and extended for about 0.5 nm. The results can be classified into four different categories:

1. A fracture event (FE) where $H_{f}$ suddenly drops at a certain bond length, accompanied by a reconfiguration of the geometric structure that is different from its initial geometry.
2. A drop in $H_{f}$ where the geometry reconfigures back to its initial structure.
3. A steady increase in $H_{f}$ during the entirety of the scan.
4. A non-convergence of the self-consistent field (SCF) calculation, where the optimal geometry of the structure is not found.

**Figure 5:**
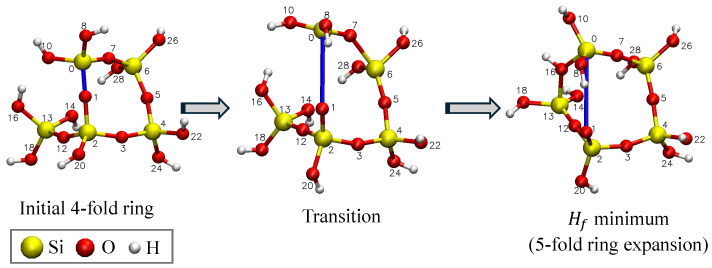
Four-ring transition to five-ring expansion with $R_{c}=0.39$ nm. This is considered to be a fracture event.

In case (1), the post-fracture structures are classified by the order of new rings formed; the initial 4-fold ring reconfigures into either a 3-fold ring (contraction), as also seen by [36], or 5-fold ring (expansion—see Figure 5). A total of 43 scans were FEs. The $a_{0}$ value for FEs is calculated using Equations (4) and (5). These $a_{0}$ values are plotted with clipping radius and fracture barrier in the following sections.

**Figure 6:**
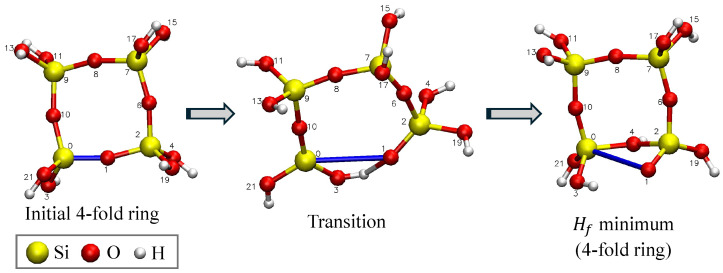
Four-ring transition to four-ring contraction with $R_{c}=0.36$ nm. Since the geometry is the same before and after, this is not considered to be a fracture event.

In case (2), the initial and final geometries after the drop in $H_{f}$ are the same. Concretely, this is seen in the 4-fold ring contraction, as seen in Figure 6, where an oxygen from one of SiO_4_ tetrahedra in the ring rotated to replace the oxygen atom that was pulled away from the ring during the scan. This configuration has a low $H_{f}$ and is, therefore, an energetically favorable configuration but does not create a free volume in the structure. Therefore, the second case is not considered to be an FE. A total of 40 scans exhibited a 4-fold ring contraction.

Case (3) refers to a total of 14 scans, where $H_{f}$ steadily rises and does not significantly drop during the entirety of the scan. In three scans, a slight drop in $H_{f}$ occurred due to the transfer of hydrogen bonds. This was not considered to be an FE as hydrogen bonds were artifacts of the simulated system, only added to neutralize the molecule.

Case (4) refers to the 55 scans that did not converge before completion. This means that self-consistency was not achieved in the geometry optimization algorithm at a certain step. This was due to the increased number of atoms considered and limitations of the theory as the potential energy landscape becomes flatter due to higher dimensionality. Non-convergence begins to appear at $R_{c}\geq 0.41$ nm in molecules with 39 atoms or more. For example, there are 66 atoms in a 0.50 nm radius sphere around one of the rings in the Bell and Dean model. Out of eight total bonds in the four-ring, only two bonds, when extended, were able to optimize the geometry at every step to the point of fracture. When the other six bonds were scanned, the calculation did not reach self-consistency after being extended only a few hundredths of a nanometer. For large structures of more than 66 atoms, non-convergence occurred for all coordinate scans, the geometry was not optimized after a small extension of the bond, and the fracture point was not identified.

### 3.2. Variation of $a_{0}$ with Clipping Radius

**Figure 7:**
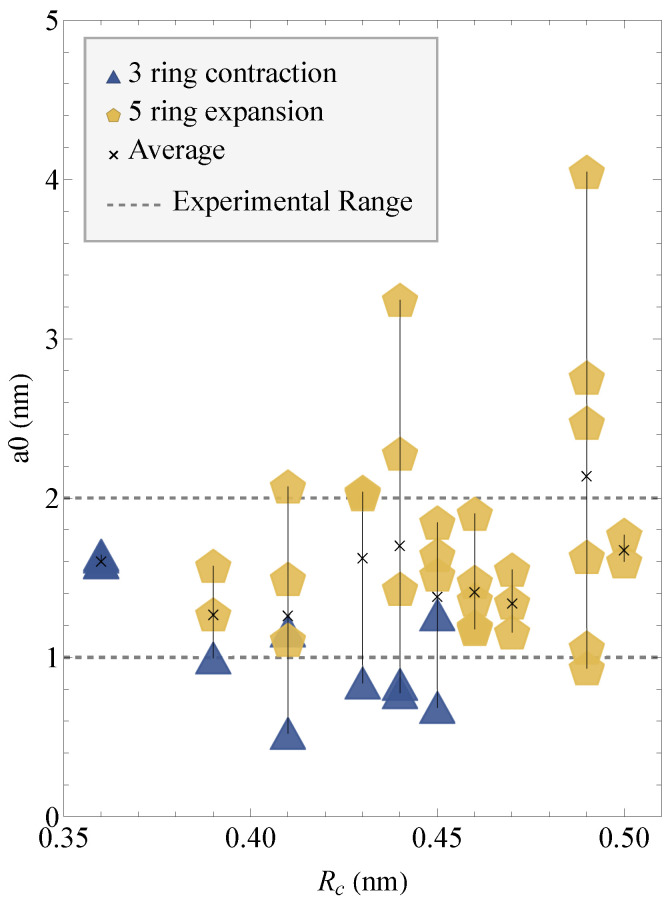
Structure parameter $a_{0}$ versus model clipping radius $R_{c}$. The data is colored by transition state structure.

The $a_{0}$ values for 3 and 5-fold FEs are plotted versus clipping radius in Figure 7. On average, the $a_{0}$ values for 3-ring contractions are less than those calculated from 5-ring expansions. The average $a_{0}$ for 3-ring contractions is 1.1±0.4 nm, with one outlier at $R_{c}=0.47$ nm and $a_{0}=82$ nm not included in the average. The average $a_{0}$ for 5-ring expansions is 1.8±0.7 nm. Overall, the total average $a_{0}$ for all fracture events is 1.6±0.7 nm. A Kruskal–Wallis test on the $a_{0}$ values for the 3-ring contractions and 5-ring expansions results in a *p*-value of 0.018, showing that the means are statistically different at the 2% level. We also calculated a linear regression for the data values to determine if there was a trend in the data. For the $a_{0}$ vs $R_{c}$ values from 3-ring contractions, the slope of the linear regression is −8.5 (no units), with R^2^ = 0.6 showing a rough downward correlation with structure size, though there are few data points to draw a conclusion. For the 5-ring expansion values, the slope is 2.8, with R^2^ = 0.018. For the entire dataset, the slope is 3.3 with an R^2^ value of 0.039, showing there is no strong correlation between the variables $a_{0}$ and $R_{c}$. From Figure 7, it is observed that there are more 3-fold ring contractions in structures with clipping radii less than 0.45 nm and more post-fracture structures with 5-fold rings as the clipping radius increases. As the 0.50 nm clipping radius is approached, there are fewer calculated $a_{0}$ values due to the limitations of the computational method.

### 3.3. Fracture and $H_{f}$ Barriers

**Figure 8:**
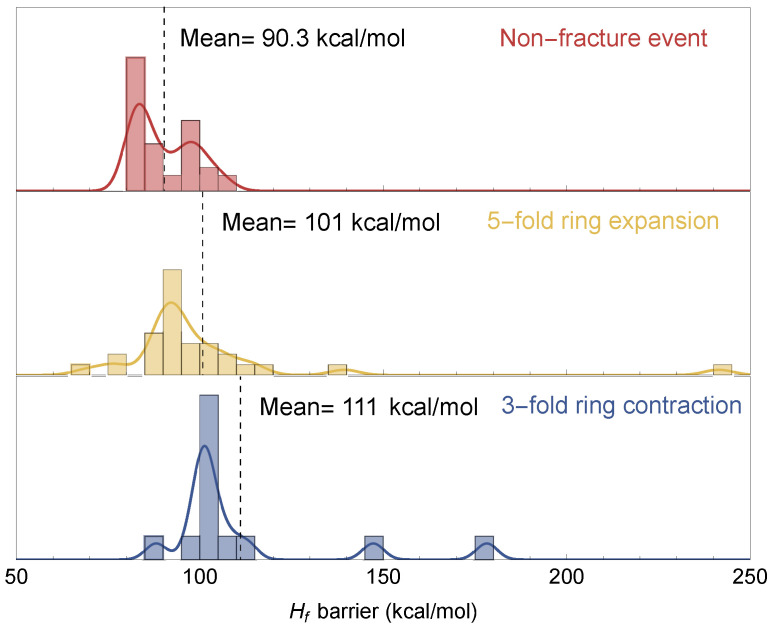
Histograms and smooth histograms showing the spread in the $H_{f}$ barrier values for non-fracture events, 3-fold ring contraction, and 5-fold ring expansion. The mean values do not include the outlier $H_{f}$ values, above 130 kcal/mol.

The fracture barriers for 3- and 5-fold ring transitions (see Section 2.4) are plotted with their corresponding $a_{0}$ values. The average fracture barriers are 102±7 kcal/mol and 94±11 kcal/mol for 3- and 5-fold ring transitions, respectively. This is greater than the $H_{f}$ barrier for case (2) non-fracture events, i.e., when a 4-fold ring transitions back into a 4-fold ring after $H_{f}$ drops, which is on average 90±8 kcal/mol. A Kruskal–Wallis test [61] with *p*-value 0.0004, which is less than 0.05, shows that the means for all three events are statistically different at the 5% level. In Figure 8, the distribution of $H_{f}$ barriers are shown for FEs and non-FEs.

### 3.4. Reconfiguration Fracture Energy

As described in Section 2.6, energy released during fracture was divided by the change in surface area of the molecule after fracture, and the values were compared to the experimental fracture energy γ. To give an example for how the reconfiguration fracture energy, $\gamma_{r}$, was calculated, we consider one structure with Rc = 0.36nm. The transition $H_{f_{t}}$ = −891.4 kcal/mol, and the minimum $H_{F_{min}}$ = −929.9 kcal/mol. The energy released during fracture is, therefore, $\Delta H_{f}=\left|H_{f_{min}}-H_{f_{t}}\right|=38.5$ kcal/mol. The conversion to kJ/molecule is 38.5 kcal/mol ×4.184 kJ/kcal $÷\;(6.02\times 10^{23}$ molecules/mol) $=2.67\times 10^{-22}$ kJ/molecule. The change in the COSMO surface area from the initial geometry to the configuration just after fracture was $\left|\Delta SA\right|=6.84\times 10^{-20}$ m^2^. Therefore, $\gamma_{r}$ is $\frac{\Delta H_{f}}{\left|\Delta SA\right|}=3.9$ J/m^2^.

Most $\gamma_{r}$ values ranged between 0.1 and 8 J/m^2^. After removing three outliers with $\gamma_{r}$ above 50 J/m^2^ (due to minor changes in the COSMO area), the mean value for all fracture events was 3±5 J/m^2^. The average $\gamma_{r}$ value for 3-fold ring contractions was about 5±4 J/m^2^, and the average $\gamma_{r}$ value for 5-fold ring expansions was about 2±6 J/m^2^.

## 4. Discussion

### 4.1. Fracture Events in a-Silica

The choice of the heat of formation as the measure of structural change should be addressed. The heat of formation usually refers to enthalpy changes during the formation of chemical compounds from elements. While the free energy is the appropriate quantity for equilibrium ensemble comparisons, our study focuses on the configurational energy landscape of a molecule that effectively identifies key rupture events and structural transitions. We use MOPAC’s built-in heat of formation as an accessible measure of the electrostatic configurational potential energy. The statistical aspect of an equilibrium quantity like the heat of formation, or associated thermodynamic quantities, implies a thermodynamic bath or an ensemble of experiments or measurements. However, the current simulations are performed quasi-statically, are not performed in a statistical ensemble, and can be considered to be done at T=0. Obviously, there will be some differences at elevated temperatures due to vibrational and entropy effects; however, the heat of formation is the same as the Gibbs free energy at zero temperature. There are other approaches to calculating the free energy available for reconfiguration [62,63], but in most cases, fluctuations are important. In the approach we have selected, we connect the reconfiguration with the fractal nature of the fracture process. By extending Si-O bonds in small sections of a-silica to the point of reconfiguration, we have observed new geometries that could lead to fracture propagation at the atomic-length scale. However, considering the statistical nature of true thermodynamic quantities, the determination of the *true* macroscopic strain energy release rate is outside of the current research scope.

There are several terms associated with the measurement of the energy at fracture. The reconfiguration fracture energy, $\gamma_{r}$ is based on the transition heat of formation minus the minimum heat of formation after fracture and should be an estimate of the molecular fracture surface energy. The mean value for $\gamma_{r}=3\pm 5$ J/m^2^ is approximately the experimental values for fracture surface energy [64]. This is different from the strain energy release rate, *G*, which is the potential energy per unit area released as the crack propagates. *G* is twice the fracture energy to create a surface as described by Griffith because there are two surfaces created [56].

In addition to the 3-fold ring contraction observed by West et al. [36] in their extension of 4-fold rings, we have observed the possibility of a 5-fold ring expansion, which also increases the strain fraction, ϵ, which is related to the free volume in the molecule. The 5-fold ring expansion usually happens when there is a dangling SiO_4_ tetrahedra bonded to the 4-fold ring of interest. When the O atom in the ring is pulled away from the Si atom, then the O atoms on the end of the singly bonded tetrahedra rotate to satisfy the valence of the Si atoms in the ring. This in turn results in the formation of a 5-fold ring. We observe that 3-fold ring contractions usually occur after longer extensions of the Si-O bond and, in general, have greater fracture barriers, and greater reconfiguration fracture energies, than 5-fold ring expansions. From a statistical mechanics perspective, a greater fracture barrier energy would make 3-fold FEs less probable than 5-fold FEs, though both events could occur during fracture. Another interpretation is that the ring’s orientation to the strain field will determine which fracture event occurs. By straining different bonds in the ring, different possible ring configurations are possible depending on the geometry of the surrounding structure. These distributions could be observed on the fracture surface using various experimental tools such as FTIR and Raman spectroscopy to determine which are the most probable configurations that lead to fracture.

### 4.2. Significance and Comparison of $a_{0}$ Calculations

Even more significant is the calculation of the parameter $a_{0}$ for both fracture events; the average value calculated for 5-fold expansions is slightly greater than for 3-fold contractions, and both fall within the experimental range of values.

From Figure 4, the calculation of $a_{0}$ depends on the distance between the Si atoms on either side of the extended Si-O bond. The average $a_{0}$ value will be greater for a lesser displacement between Si atoms after fracture. For 5-fold ring expansions, where the two Si atoms are still in the ring, $a_{0}$ is greater than that of 3-fold ring contractions, where one of the Si atoms is removed from the ring and, therefore, has a greater displacement.

In our simulations, the average value for $a_{0}$ from the 3 and 5-ring configurations is 1.6±0.7 nm, which is within the experimental range for amorphous silica and silicate glasses, between 1.0 and 2.0 nm [22]. The value we calculated is also within the range of the $a_{0}$ value reported in [1], 1.14 nm. The difference could arise from a few factors. First, the software that was used to calculate the initial value has been updated in the past 20 years. When we simulated the same isolated 4-fold ring structures as [36] using AM-1 theory, the fracture barriers that we calculated for 3-fold ring contractions was greater by 19 to 36 kcal/mol and ring contraction only occurred after the Si-O bond was extended at least 0.339 nm to 0.50 nm as compared to the previously reported value of 0.310 nm. Next, our work extends that of West et al. [1,36] with the simulation of larger structures. With more atoms surrounding the ring in a network, there are more possibilities for rearrangement. From the calculations of $a_{0}$ for different structure sizes, a larger spread in $a_{0}$ was observed than that reported in [1]. Finally the speed of the calculations in 2024 is much faster than it was thirty years ago. West et al. [1,36], would run a single simulation over the weekend, while we can run a single coordinate scan in about 12 min on a supercomputer. This allows us to perform more trials and get a variety of results. There is no randomness in the calculations themselves; a given input file will give the same output each time the calculation is performed. However, there are different ways to set up the simulation. We performed different trials by separately scanning each of the eight Si-O bonds in the same ring. By performing these calculations, we observe at least three possibilities with the isolated 4-fold ring: 3-fold contraction due to the removal of either an O atom or Si atom from the ring and 4-fold contraction with a hydrogen defect. The range of post-fracture structures produces a range of $a_{0}$ values.

Despite the variety in $a_{0}$ values calculated, we observe that the average $a_{0}$ does not correlate with structure size within the limits of this present study. We observe this even though the larger structures have more 5-ring expansions, and the smaller structures have more 3-ring contractions.

### 4.3. Relation to the Macroscopic-Length Scale

The invariability of the average $a_{0}$ value with structure size would be the evidence that fractal analysis links fracture behavior at the atomic and macroscopic scale. We envision that the small number of atoms that we are using for calculations represents various possible geometrical configurations and orientations of rings all along the crack front. When the strain at the crack tip triggers the reconfiguration that we observe, then this change in structure results in an increased strain in the nearby ring structures that further results in more reconfigurations and an increase in crack extension. Since the structures are randomly oriented, these reconfigurations all along the crack front result in a fracture surface structure that is not smooth [65,66], as observed with AFM measurements [67,68]. The fractal dimensional increment, $D^{*}$, can be measured on the tortuous fracture surface. $D^{*}$ is also related to $a_{0}$ and to fracture toughness, $K_{IC}$, or fracture energy γ via Equations (2) or (3), respectively.

### 4.4. Limitations

One of limiting factors for these simulations of fracture is the number of atoms in the structure. Semi-empirical molecular orbital (SE-MO) theories such as AM-1 are able to efficiently handle more atoms than DFT at the expense of some approximation. A second limiting factor is the ability of SE-MO theories to model long-range atomic interactions. AM-1 does not include the wave functions for unpaired electrons, which would give more accurate results for bond breaking. However SE-MO simulations, which are also used to find transition states for chemical reactions, still give an idea for what equilibrium structures at energy minima are possible after fracture, when atoms have re-bonded. The small size of the molecules that we have simulated gives us a glimpse at the possible reconfiguration structures at the crack front, even if the transition state structures or energy barriers are not entirely accurate. It is promising to see that the fact that the calculations of $a_{0}$ using these methods are within the range of experimental values. Though calculations can be made more robust, this research provides evidence that it is possible to calculate $a_{0}$ at the atomic level. It also provides evidence that $a_{0}$ can be measured from atomic ring reconfiguration, especially seeing that its value is similar for both 3-fold ring contraction and 5-fold ring expansion of the ring.

Currently, molecular orbital (MO) simulations in general are limited in their ability to simulate large structures, but our approach strikes a balance between chemical fidelity and computational feasibility. One alternative approach would be to use molecular dynamics (MD). However, MD can handle large systems at the expense of missing quantum effects. Some potentials, such as ReaxFF [69], have been developed to approximate the effects of bond breaking and formation but have not been proven for large reconfigurations. At the other end of the spectrum, we could attempt doing all simulations through DFT. Unfortunately, even at small system sizes, the calculations become computationally unreasonable and do not converge. Another possible route for simulating larger structures is to use MD [70] to the point just before bond reconfiguration and then use MO and DFT calculations to more accurately find the resulting structure after fracture. This might be a viable approach and is the subject of current research, but smaller reconfigurations (as reported in this paper) will be needed to test such approaches. As improvements in quantum chemistry simulations develop, future investigations of the calculations of $a_{0}$ should ultimately involve the simulation of a surface of material with enough atoms to reproduce the tortuosity seen on fracture surfaces. This would demonstrate more clearly the link between $a_{0}$ measured from ring contraction or expansion and calculations from the macroscopic measurement of the fractal dimensional increment of the fracture surface.

## 5. Conclusions

1. We have successfully extended the work of West et al. [1,36], by using a CRN model to simulate fracture in larger molecular structures found in a-silica. The larger structures are assumed to more closely resemble the true environment of the ring in a glass structure embedded in an extensive network of atoms.

2. We propose that a fracture in amorphous silica occurs due to bond reconfiguration resulting in an increased strain volume at the crack tip.

3. We hypothesize two specific configurations that lead to a crack extension from a four-fold ring reconfiguration to three-fold ring or five-fold ring configurations resulting in a change in volume as characterized by the parameter $a_{0}$. The 5-fold ring contraction was a new discovery not observed in West et al. [36].

4. In amorphous silica, $a_{0}$ can be considered an indirect measure of the free volume available or created at the time of fracture, where there is a new structural rearrangement and not just a bond separation. This finding is critical to understanding the fractal nature of fracture as a discontinuous process.

5. $a_{0}$ can be computed from macroscopic experimental measurements of toughness, elastic modulus, and fractal dimension without using adjustable constants. We have shown a computational method that gives $a_{0}$ values that have the same order of magnitude as experimental $a_{0}$ values.

6. We developed a technique to compare the experimental fracture energy at the macroscopic scale to a reconfiguration fracture energy at the atomic-length scale. The ability to predict the fracture properties of macroscopic materials by looking at their atomic structure can improve the way that materials are designed from first principles. The glass industry will benefit from using simulations to guide the development of new materials as opposed to an extensive experimental methodology.

## Data Availability

The original contributions presented in this study are included in the article. Further inquiries can be directed to the corresponding author.

## References

[1] West J., Mecholsky J., Hench L. (1999). The application of fractal and quantum geometry to brittle fracture. J. Non-Cryst. Solids.

[2] Sun Y., Edwards M.G., Chen B., Li C. (2021). A state-of-the-art review of crack branching. Eng. Fract. Mech..

[3] Bitzek E., Kermode J.R., Gumbsch P. (2015). Atomistic Aspects of Fracture. Int. J. Fract..

[4] Jiang Y., Killough J.E., Cui Y. (2022). A numerical simulation approach for shale fracture network characterization using hybrid EDFM method. Lithosphere.

[5] Yan M., Wu D. (2023). A New Fracture Simulation Algorithm Based on Peridynamics for Brittle Objects. IEEE Access.

[6] Zhou Z., Su Y., Wang W., Yan Y. (2017). Application of the fractal geometry theory on fracture network simulation. J. Pet. Explor. Prod. Technol..

[7] Lan M., He Y., Wang C., Liu X., Ren G., Zhang S. (2024). Fractal evolution characteristics of fracture meso-damage in uniaxial compression rock masses using bonded block model. Sci. Rep..

[8] Wang W., Su Y., Zhang Q., Xiang G., Cui S. (2018). Performance-based fractal fracture model for complex fracture network simulation. Pet Sci..

[9] Yangsheng Z., Zengchao F., Dong Y., Weiguo L., Zijun F. (2015). Three-dimensional fractal distribution of the number of rock-mass fracture surfaces and its simulation technology. Comput. Geotech..

[10] Mecholsky J., DeLellis D., Mecholsky N. (2020). Relationship between fractography, fractal analysis and crack branching. J. Eur. Ceram. Soc..

[11] Mandelbrot B.B. (1982). The Fractal Geometry of Nature.

[12] Mandlebrot B., Passoja D., Paulley A. (1984). Fractal character of fracture surface of metals. Nature.

[13] Mecholsky J.J., Passoja D.E., Feinberg-Ringel K.S. (1989). Quantitative Analysis of Brittle Fracture Surfaces Using Fractal Geometry. J. Am. Ceram. Soc..

[14] Russ J.C. (1994). Fractal Surfaces.

[15] Mackin T., Mecholsky J. (1987). A Fractal Analysis of Brittle Fracture.

[16] Williford R. (1988). Multifractal fracture. Scr. Metall..

[17] Mecholsky J.J., Freiman S.W. (1991). Relationship between Fractal Geometry and Fractography. J. Am. Ceram. Soc..

[18] Mosolov A.B. (1993). Mechanics of Fractal Cracks in Brittle Solids. Europhys. Lett..

[19] Milman V.Y., Stelmashenko N.A., Blumenfeld R. (1994). Fracture surfaces: A critical review of fractal studies and a novel morphological analysis of scanning tunneling microscopy measurements. Prog. Mater. Sci..

[20] Hill T., Mecholsky J., Anusavice K. (2000). Fractal Analysis of Toughening Behavior in 3BaO5SiO_2_ Glass-Ceramics. J. Am. Ceram. Soc..

[21] Santos S., Rodrigues J. (2003). Correlation Between Fracture Toughness, Work of Fracture and Fractal Dimensions of Alumina-Mullite-Zirconia Composites. Mater. Res..

[22] Mecholsky J. (2006). Estimating theoretical strength of brittle materials using fractal geometry. Mater. Lett..

[23] Chang Q., Chen D.L., Ru H.Q., Yue X.Y., Yu L., Zhang C.P. (2011). Three-dimensional fractal analysis of fracture surfaces in titanium–iron particulate reinforced hydroxyapatite composites: Relationship between fracture toughness and fractal dimension. J. Mater. Sci..

[24] Sahu S., Yadav P.C., Shekhar S. (2017). Fractal Analysis as Applied to Fractography in Ferritic Stainless Steel. Metallogr. Microstruct. Anal..

[25] Yarema S.Y. (1996). On the contribution of G. R. Irwin to fracture mechanics. Mater. Sci..

[26] Sanford R. (2003). Principles of Fracture Mechanics.

[27] Hilders A., Pilot D. (1997). On the Development of a Relation between Fractal Dimension and Impact Toughness. Mater. Charact..

[28] Key W.B., Jodha K.S., Kaur N., Salazar Marocho S.M., Mecholsky J.J., Griggs J.A. (2022). Fracture toughness and fractal analysis of ceramic benchmark materials. J. Mater. Sci..

[29] Lange D.A., Jennings H.M., Shah S.P. (1993). Relationship between Fracture Surface Roughness and Fracture Behavior of Cement Paste and Mortar. J. Am. Ceram. Soc..

[30] Lung C.W., Mu Z.Q. (1988). Fractal Dimension Measured with Perimeter-area Relation and Toughness of Materials. Phys. Rev. B.

[31] Mecholsky J.J., Mackin T.J. (1988). Fractal analysis of fracture in Ocala chert. J. Mater. Sci. Lett..

[32] Carney L.R., Mecholsky J.J. (2013). Relationship between Fracture Toughness and Fracture Surface Fractal Dimension in AISI 4340 Steel. Mater. Sci. Appl..

[33] Mecholsky J.J., Mackin T.J., Passoja D.E., Varner J., Frechette. V.D. (1988). Self-similar crack propagation in brittle materials. Advances in Ceramics: Fractography of Glasses and Ceramics.

[34] Freiman S.W., Mecholsky J.J. (2019). The Fracture of Brittle Materials: Testing and Analysis.

[35] Tsai Y., Mecholsky J. (1991). Fractal fracture of single crystal silicon. J. Mater. Res..

[36] West J., Hench L. (1994). Silica fracture: Part 1 A ring contraction model. J. Mater. Sci..

[37] Dewar M., Zoebisch E., Healy E., Stewart J. (1985). AM1: A New General Purpose Quantum Mechanical Molecular Model. J. Am. Chem. Soc..

[38] Bell R., Dean P. (1972). The structure of vitreous silica: Validity of the random network theory. Philos. Mag..

[39] Galeener F.L. (1982). Planar Rings in Vitreous Silica. J. Non-Cryst. Solids.

[40] Galeener F., Barrio R., Martinez E., Elliott R. (1984). Vibrational Decoupling of Rings in Amorphous Solids. Phys. Rev. Lett..

[41] Wright A.C., Thorpe M.F. (2013). Eighty Years of Random Networks. Phys. Status Solidi.

[42] Humphrey W., Dalke A., Schulten K. (1996). VMD—Visual Molecular Dynamics. J. Mol. Graph..

[43] Collins M.A. (2009). The interface between electronic structure theory and reaction dynamics by reaction path methods. Advances in Chemical Physics.

[44] Lewis L.J. (2022). Fifty years of amorphous silicon models: The end of the story?. J. Non-Cryst. Solids.

[45] Mecholsky N.A., Morin R., Mecholsky R.D., Freiman S.W., Mecholsky J.J. (2024). Dataset from the Bell and Dean paper on the structure of vitreous silica. Data Brief.

[46] Ishimaru M., Munetoh S., Motooka T. (1997). Generation of amorphous silicon structures by rapid quenching: A molecular-dynamics study. Phys. Rev. B.

[47] Zheng Z., Zheng F., Wu Y.N., Chen S., Wang L.W. (2025). A Generalized Bond Switching Monte Carlo method for amorphous structure generation. Comput. Mater. Today.

[48] Ding Z., Zhou S., Zhao Y. (2004). Hardness and fracture toughness of brittle materials: A density functional theory study. Phys. Rev. B.

[49] Sakanoi R., Shimazaki T., Xu J., Higuchi Y., Ozawa N., Sato K., Hashida T., Kubo M. (2014). Communication: Different behavior of Young’s modulus and fracture strength of CeO_2_: Density functional theory calculations. J. Chem. Phys..

[50] Yuan Z., Zhang C., Li L., Xu X., Wang X. (2020). Density functional theory calculation of fracture surfaces of siderite and hematite. Powder Technol..

[51] Jung G.S., Irle S., Sumpter B.G. (2022). Dynamic aspects of graphene deformation and fracture from approximate density functional theory. Carbon.

[52] Felix L.C., Li Q.K., Penev E.S., Yakobson B.I. (2025). Ab Initio Molecular Dynamics Insights into Stress Corrosion Cracking and Dissolution of Metal Oxides. Materials.

[53] Zhang H., Sun W., Xie X., He J., Zhang C. (2023). Insights into the Fracture Nature of Hematite from First Principles DFT Calculations. ACS Omega.

[54] Lazar P., Podloucky R. (2008). Cleavage fracture of a crystal: Density functional theory calculations based on a model which includes structural relaxations. Phys. Rev. B.

[55] Vashishta P., Kalia R.K., Nakano A., Yip S. (2005). Multimillion Atom Molecular-Dynamics Simulations of Nanostructured Materials and Processes on Parallel Computers. Handbook of Materials Modeling: Methods.

[56] Griffith A. (1921). The phenomenon of rupture and flow in solids. Philos. Trans. R. Soc. Lond. Ser. Contain. Pap. Math. Phys. Character.

[57] Irwin G. (1961). Fracturing and Fracture Mechanics, T&AM Report No 202.

[58] Weser O., Hein-Janke B., Mata R.A. (2023). Automated handling of complex chemical structures in Z-matrix coordinates—The chemcoord library. J. Comput. Chem..

[59] Mecholsky J.J., West J.K., Passoja D.E. (2002). Fractal dimension as a characterization of free volume created during fracture in brittle materials. Philos. Mag. A.

[60] Klamt A., Schüürmann G. (1993). COSMO: A new approach to dielectric screening in solvents with explicit expressions for the screening energy and its gradient. J. Chem. Soc. Perkin Trans. 2.

[61] Kruskal W.H. (1952). A Nonparametric test for the Several Sample Problem. Ann. Math. Stat..

[62] Vega C., Noya E.G. (2007). Revisiting the Frenkel-Ladd method to compute the free energy of solids: The Einstein molecule approach. J. Chem. Phys..

[63] Eslami H., Müller-Plathe F. (2024). Metadynamics simulations of three-dimensional nanocrystals self-assembled from triblock janus nanoparticles: Implications for light filtering. ACS Appl. Nano Mater..

[64] Wiederhorn S.M. (1969). Fracture Surface Energy of Glass. J. Am. Ceram. Soc..

[65] Beauchamp E.K., Varner J., Frechette V., Quinn G. (1996). Mechanisms for hackle formation and crack branching. Proceedings of the Ceramic Transactions: Fractography of Glasses and Ceramics III.

[66] Guilloteau E., Arribart H., Creuzet F. (1995). Fractography of Glass at the Nanometer Scale. MRS Online Proc. Libr..

[67] Smith R., Mecholsky J. (2011). Application of atomic force microscopy in determining the fractal dimension of the mirror, mist, and hackle region of silica glass. Mater. Charact..

[68] Garza-Méndez F., Hinojosa-Rivera M., Gómez I., Sánchez E. (2007). Scaling properties of fracture surfaces on glass strengthened by ionic exchange. Appl. Surf. Sci..

[69] van Duin A.C.T., Dasgupta S., Lorant F., Goddard W.A. (2001). ReaxFF: A Reactive Force Field for Hydrocarbons. J. Phys. Chem. A.

[70] Wu P., Liu R., Li W., Zhang W., Wei J., Zhou Q., Wei T., Kardani A., Lin Z., Xiao Y. (2025). Interface optimization by introducing Ti for strengthening graphene network/copper composites: New insight from MD simulations. Carbon.

